# The tobacco industry’s thwarting of marketing restrictions and health warnings in Lebanon

**DOI:** 10.1136/tc.2008.029405

**Published:** 2009-07-17

**Authors:** R Nakkash, K Lee

**Affiliations:** 1 Department of Health Behaviour and Education, Center for Research on Population and Health, American University Beirut, Beirut, Lebanon; 2 Centre on Global Change and Health, London School of Hygiene and Tropical Medicine, Keppel Street, London WC1E 7HT UK

## Abstract

**Aims:**

This article outlines how the tobacco industry has undermined tobacco control efforts in Lebanon since the early 1970s.

**Methods:**

An analysis of online and on-site tobacco industry documents, reviews of newspapers, policy and other documents, and interviews with key policy makers were conducted.

**Results:**

Findings reveal how the weakness of tobacco control legislation in Lebanon has been the product of an effective tobacco industry strategy to weaken the content and scope of regulation, and delay adoption and implementation.

**Conclusions:**

The tobacco industry has built and maintained strong alliances that were and are regularly mobilised to effectively oppose regulation.  Despite ratification of the World Health Organization Framework Convention on Tobacco Control in 2005, Lebanon's tobacco control track remains weak. Public health professionals and the government should work hard to oppose such tobacco industry tactics.

Adult cigarette smoking prevalence in Lebanon remains relatively high at 29% for males and 6.9% for females, the highest in the Middle East after Jordan.^1^ Cancer morbidity and mortality trends reflect a smoking epidemic in its mature stages.^2^ ^3^ Overall, tobacco is the leading cause of preventable deaths (3500 deaths annually),^4^ predicted to kill more people over the next 30 years than the 16-year civil war.^5^

Despite this health burden, tobacco control in Lebanon remains relatively weak.^1^ Saudi Arabia, Oman, Yemen, Bahrain, Morocco, Qatar, Iran, Libya, United Arab Emirates, Egypt, Kuwait, Syria and Jordan have all adopted advertising restrictions. All have full or partial public smoking bans, although enforcement is variable. Almost half have increased taxes as a policy measure.^6^ In contrast, Lebanon has no national tobacco control policy, no restrictions on direct advertising, promotion or sponsorship and no law on smoke-free environments.^1^

The policy influence of the tobacco industry in the Middle East has been previously analysed largely based on Philip Morris (PM) documents. Focusing on the Middle East Working Group (MEWG) and Middle East Tobacco Association (META), the reports describe the undermining of smoking restrictions, cigarette testing procedures and taxation in the Gulf countries and Saudi Arabia.^7^ ^8^

This paper analyses how the tobacco industry has undermined tobacco control in Lebanon from the 1970s to early 2000s. By analysing documents of British American Tobacco (BAT), PM and RJ Reynolds (RJR), supported by key informant interviews, industry efforts to weaken marketing restrictions and health warnings are described. The authors have previously argued that transnational tobacco companies (TTCs) have pursued market access to Lebanon^9^ because of its significance as a regional “trendsetter”.^10^ For similar reasons, the industry has sought to delay or weaken marketing restrictions and warning labels. These findings suggest the need for greater transparency in the role of the tobacco industry in public policy making, as part of the government’s commitments under the World health Organization (WHO) Framework Convention on Tobacco Control (FCTC).

## METHODS

A total of 65 files related to the Middle East were reviewed at the BAT Guildford Depository in 2004. From 2005–2008, document searches were undertaken of the BAT Document Archive (BATDA)^11^ ^12^ and Legacy Tobacco Document Library. Keywords used related to industry personnel, organisations, policy makers, government officials and specialist industry terms. Alternative spellings and Boolean searches were used to combine keywords. A total of 2340 documents were reviewed which enabled the construction of a historical and thematic narrative. Further analysis incorporated validation techniques within a hermeneutic approach^13^ including corroboration of interpretation between authors.

Official documents of the state-owned monopoly, the Regie (*Regie Libanaise des Tabacs et Tombacs*) and Ministry of Public Health are not publicly accessible. Thus, 20 semistructured interviews were conducted in Lebanon in 2006 with key informants including public officials, industry representatives and public health advocates. Interviews, recorded and transcribed in Arabic and then translated into English, supported the interpretation of industry documents by contextualising and confirming their content. Transcripts were coded by keyword to facilitate their use alongside documents. Secondary sources, including local media reports, scholarly works and industry sources, were used as supplementary data sources.

## RESULTS

### Marketing and advertising restrictions “will be shelved”

Efforts to restrict tobacco marketing and advertising in Lebanon began in 1970 when Minister of Public Health (MOPH) Moutran Habib introduced draft legislation on cinemas and television.^14^ The industry’s ally in opposing the bill was the advertising sector. Advertising executive Muhamed Shukayr (General Manager, Societe Orientale de Publicite) reassured William Dales (PM Overseas Office, Greece) that “many factors…will prevent execution of the Minister’s order” including a predicted adverse effect on “the plantation of tobacco, production of cigarettes and trade”.^14^ Also supporting the industry, according to former director of the Regie Walid Salam were local agents and distributors with interests in other sectors:

These distributors are not only distributors of tobacco, like Marlboro…Kettaneh [Freres, PM’s local distributor] has a million other things, like construction, electricity, etc. They don’t pressure specifically for tobacco but they can bother the government in other things so the government is forced to listen to them in the end. (Interview; Beirut, 22 May 2006)

Lobbying with Kettaneh Freres, Shukayr correctly predicted “the matter will be shelved”.^14^ A second bill introduced in 1973, to ban advertising of foreign cigarettes^15^ met a similar fate.

With the outbreak of civil war in 1975, there were no further developments until the Lebanese Cancer Society’s anti-tobacco campaign in 1978.^16^ PM reported “periodic coverage in the Lebanese press, which is liberal in character and holds a predominant position in the pan-Arab region”.^16^ Anti-smoking sentiment, endorsed by the Arab League^17^ ^18^ was thought to mainly exist among “the highly educated and the medical profession…influenced by…Western Europe and in America”.^16^

Seeking “to maintain the ability to advertise our products in a convincing and cost effective manner”,^17^ PM again found an ally in the advertising industry. In April 1980, Shukayr argued to MOPH Talal Miri’bi and Prime Minister Salim El Hoss that “prompt execution of the decree—as far as the advertising requirements are concerned—would give way to transfer of pan Arab advertising budgets to other countries”.^19^ According to Shukayr, both “promised [to] take the necessary step to defer the decree’s execution till it is properly restipulated”.^19^ Advertising agencies followed up with “a memo to the minister, a copy to the Regie, detailing this difficulties [sic] of applying it immediately and asking for a normal delay”.^19^ In May 1980, Macleod reported to Walter Thoma (PM International) that:

…the decree has been suspended and a joint committee composed of members of the Regie and the Ministry of Health to be formed to study the proposals. Mr Mohamed Shukayr, who is both PM advertising agent and President of the Lebanese Advertising Association, intends to request the participation of the Association in the joint committee.^18^

Limited progress over the next decade suggests industry’s opposition was effective. There was little further discussion of marketing restrictions until 1994 when MOPH Marwan Hmade revived the issue, insisting that health would be placed over profits.^20^ Later reflecting on his efforts, Hmade cited the advertising industry as his key opponent: “In Parliament, whenever we attempted to go further on banning tobacco advertising…we always clashed with the lobbyists. I would not say the tobacco lobby but the tobacco *advertising* lobby” (interview; Beirut, 31 May 2006). Pressure by advertising agencies, according to former National Tobacco Control Programme (NTCP) manager Youssef Bassim, led to a television presenter being threatened with his job if the MOPH was asked about tobacco issues.^21^ More subtly, Nadine Keirooz (Director, Tobacco Free Initiative Lebanon) describes how the media was unreceptive to giving tobacco control issues “visibility” (interview; Beirut, 29 June 2006). Mroeh, (former MOPH) believes the global scale of media interests created political influence:

The TV and all the media, not only newspapers, but the most important are the advertising companies… which get money from the tobacco companies.…The tobacco companies, of course, feed into huge international companies that give locally but have huge businesses in London, New York. (Interview; Beirut, 11 May 2006)

### Voluntary codes: “[P]ut the matter to bed…for many years to come”

Alongside lobbying, the industry supported a voluntary code in Lebanon to delay or prevent binding regulation.^22^^–^25 Following a meeting in 2000 of Michael Erickson (US Centers for Disease Control and Prevention), Lawrence Green (Director for Smoking or Health) and MOPH Karam Karam to discuss a proposed advertising ban, Najjar and Maher Achi (Chief Operating Officer, Leo Burnett) planned “informal” approaches to the MOPH on a social occasion to suggest youth smoking prevention programmes. The strategy was:

…to consider internally and jointly with the rest of the industry, those marketing practices that we may be willing to concede, preferably voluntarily (and eventually revise the industry’s voluntary marketing code to reflect such concessions). Alternatively, such concessions may become necessary under threat of legislative amendment, and we should be prepared with our list of reasonable concessions….

[this is] an opportunity to…constructively engage the Ministry on youth smoking prevention, to mobilise allies and hopefully put the matter to bed in a manner satisfactory to all parties for many years to come.^22^

Previously, in August 1995 Charles Hay (META Dubai) wrote to members on a meeting:

[t]o devise and agree an internal ‘voluntary code of marketing practice’ that clarifies all marketing freedoms not already proscribed and that can be used as a demonstration of responsibility with Government in the event of further proposed legislation….^10^

A draft Voluntary Marketing Code for Lebanon was circulated in October 1995 (and approved in 1996)^26^^–^29 with the preamble:

(a) that cigarettes are a legally traded product;

(b) that smoking is an adult activity and that any advertising’ or promotional activity undertaken by the industry will only be designed for and marketed to adult smokers that are defined as smokers of 18 years or older;

(c) that adults who choose to smoke are entitled to information on existing and new brands and styles of cigarettes;

(d) that advertising is an important means of communication with consumers and is necessary to maintain fair competition between brands and that advertising is an essential component of a free market economy…

Print: no branded cigarette advertising shall be specifically directed at minors. No branded cigarette advertising shall appear in print media which is directed principally toward minors.^27^

According to George Saade (former director of the National Tobacco Control Program), however, advertisers were reluctant to accept even voluntary restrictions: “The media companies are blackmailing these big multinationals in Lebanon telling them, “Listen you are losing market share here if you don’t do ads because other companies are doing advertising and market share is going up”. Saade describes how TTCs approached him for support:

BAT and PM… told me that they want to stop the advertising of tobacco products but they need backup from the MOPH and Lebanese government. They said, ‘We will back up the Lebanese government to ban ads on tobacco products but we know that media companies are against us on this proposal’. (Interview; Beirut, 15 March 2006)

According to Ramsay Najjar (former President of the Advertising Association), protecting market share was the real motive of TTCs:

If they close advertising now, the newcomers cannot advertise and thus take their market share. So they stopped the game at a time when they had the highest market share in the market. That’s why the first who started this idea and started convincing others to stop advertising also – what they called ‘responsible marketing’, the buzz word of corporate multinationals…the first who demanded it was PM, whom are market leaders worldwide. (Interview; Beirut, 15 June 2006)

Chair of the Parliamentary Committee for Public Health, Labour, and Social Affairs (PHC) Atef Majdalani agrees:

…big companies…don’t care about advertising since they are already known and don’t care about it. Not because they are Caritas [a Lebanese social welfare foundation], but because there are new companies entering the market. If advertising stops, it hurts the new companies coming into the market since everybody already knows existing brands. (Interview; Beirut, 3 March 2006)

Given the dominance of PM and BAT, legislation to ban “above the line” (direct) advertising would be like “coming to put out the fire, when all has turned to ashes” (interview; Beirut, 15 June 2006). As shown elsewhere,^30^ ^31^ PM and BAT then shifted to “below the line” (indirect) activities not subject to the voluntary code, such as sponsorship and brand stretching. The strategic success of the voluntary code to prevent binding regulation appears affirmed by the failure of draft legislation in October 1998 to progress beyond the Council of Ministers.^32^

### To “temporarily freeze the implementation of” health warnings

Health warnings have been the subject of the only two pieces of tobacco control legislation in Lebanon to date. Documents suggest that, as well as limiting their scope, the industry effectively undermined their implementation.

The first two attempts to introduce health warnings came in 1976 and 1978, with both failing to progress beyond a draft bill. No warning size was specified and the proposed wording was relatively weak: “The Lebanese Ministry of Health warns you of the hazards of smoking”.^33^ A stronger message, “Smoking is the main cause of lung cancer, cardiovascular disease and arterial disease”, was proposed in 1980,^33^ which would occupy 10% of the packet area of domestic and imported brands. Kettaneh Freres wrote to Macleod objecting that the decree was “without consultation with interested parties”, “strict and arbitrary” and would have negative economic repercussions.^34^ ^35^

While PM noted that several bills “have not been implemented due to the security situation”,^18^ growing regional support for tobacco control warranted attention. Towards this end, it initiated lobbying which it “alone should direct” as “[d]iscussion and coordination with the rest of the industry will only confuse and delay action…we have all the necessary influence and contacts”.^36^ While taking the lead in seeking “modification of abusive clauses”.^37^ PM maintained a low profile to create the impression that lobbying was by local interests:

The [lobbying] file should be compiled primarily to include scientific information, which can be used against qualified medical opinion from the Lebanese Ministry of Health. I am still very much in favour of the idea of getting the relevant Regie managers to attend a presentation and our agent in Lebanon will keep the pressure on this proposal. However, in the absence of this action, believe an anonymous (not PM) file of information to be of value.^37^

Economic arguments were again invoked: “Lebanon is the most important source of Pan Arab press coverage. Unreasonable warnings on advertising might encourage advertisers to reduce their advertising in Lebanese publications, in favour of sources where less obnoxious warnings apply”.^18^ In June 1980 Kettaneh Freres updated Macleod on lobbying efforts:

The Regie…hopes to temporarily freeze the implementation of this regulation and requests that, in the case of a new study, it be involved in a committee of mixed membership that would define the principles of the new regulation. It is certain that, at this initial stage, the action taken by the Regie constitutes a positive step; however, should the Minister of Public Health, under the influence and with the help of the scientific circles of AUB [American University of Beirut], go back and discuss the same problem, the Regie would be left with no scientific basis with which to defend itself in this issue [translated from French].^34^

Macleod, in turn, told Richard Corner (International Advertising Association) that there was little cause for concern since “none of the [previous] provisions were ever implemented”.^38^

Implementation was delayed for several years further. One reason was variation in warnings across the region.^39^ In Saudi Arabia, for example, pan-Arab publications contained the phrase, “Government warning: smoking harms your health and we advise you to refrain from it”.^40^ This was stronger than the phrasing in Lebanon, but weaker than Saudi regulations.^40^ Documents suggest the industry used this confusion to push for non-compliance. With the adoption of the 1983 law in Lebanon, for example, the MEWG reported that “Advertisements in Pan Arab Press printed outside the Lebanon, would not carry Warnings”.^41^

Another issue was timing. Martin Canon (BAT) wrote to Kirkland Blair (Rothmans) in 1981 stating that “we have general agreement not to ship new health warning stock to any markets unless there has been a published decree”.^42^ There was also concerns health warnings could be “construed as an admission of causation by the industry”.^43^ BAT’s US subsidiary Brown and Williamson (B&W) opposed referring to “specific diseases” or attributing the warning to an official body such as government.^44^ ^45^ An added complication was the contraband trade which supplied “[o]ver 50% of the cigarette sales in Lebanon” without warning labels.^10^

According to Richard Davies (Public Affairs, BAT), PM supported health warnings to pre-empt stronger regulation and “commercial advantage”.^46^ As documented elsewhere,^47^ PM “would ‘negotiate’ the introduction of health warnings where none currently exist on the basis that they might be able to achieve ‘Mild’ versions”.^48^ Patrick Sheehy (Chairman, BAT) noted PM’s potential economic advantage: “It has been rumored that PMI instigated the decision to require a health warning…Apparently, PMI will reduce the price of MARLBORO once the health warning is implemented”.^48^

Documents suggest that, even after taking effect, efforts to undermine the law continued. A MEWG meeting reported that “Lebanon R.J.R. tabled a draft Decree and English translation…that after amendment it had been signed by the Prime Minister and was effective from 16.10.83”.^49^ Along with the removal of tar and nicotine limits, the size of warnings was further limited to 5% of the pack size. Because of warnings in “unreadable fine print” (Mroeh interview; Beirut, 11 May 2006), the Council of Ministers amended the decree in December 1983 to 10% of pack size (FCTC provisions later adopted recommend no less than 30% and optimally 50%). A 1984 MEWG meeting noted unsuccessful industry efforts to overturn the law: “[w]hether the new Decree could be frozen or not was out of our control”.^49^ Attention instead focused on maintaining weak phrasing, a tactic successfully used elsewhere^50^ Moreover, warnings scuppered long-anticipated restrictions on advertising:

They went to the President… [who] called me and told me this rule will create problems with the newspapers and how we can’t let them lose money and go bankrupt…So let’s adjust it a bit. Therefore we reduced from a total ban on advertising to a warning. (Mroeh interview; Beirut, 11 May 2006)

More effective implementation was not pursued further until after the civil war.^51^ ^52^ In 1992, the PHC began discussing stronger warnings, such as “Smoking causes cancer, heart and artery disease”, which would be rotated, occupy 20% of pack size and extend to broadcast media. The amendment would also allow the MOPH to follow tobacco advertisements with a counter advertisement.^53^ ^54^ Documents suggest intense industry lobbying again delayed and weakened the proposed revision: “[f]ollowing discussion with the tobacco companies and advertising agencies, the government has made no moves to introduce it. Industry/government talks on an ongoing informal basis aim to slow down the possible introduction of further legislation”.^55^ The amendment, eventually adopted in 1995, used the phrase, “The Ministry of Health Warns: Smoking Leads to Serious and Fatal Diseases”.^56^ Instead of a specific size, the law required pack warnings to be visible to the naked eye, 15% of print advertisements and 15% of broadcast media. There was no provision for rotating warnings.

On World No Tobacco Day in June 2000, MOPH Karam announced his intention to ban tobacco advertisement.^57^ He proposed to expand “the space used for health warnings on cigarette packages to 60% of the surface area”.^58^ While two bills were proposed in 2003 and 2004, none have been adopted to date.

### The PHC: “putting up barriers and obstacles”

The Lebanese government’s failure to adopt stronger tobacco control since 2000 has been due in part to renewed political and economic instability. The lack of a functioning government and public administration, amid simmering conflict and sectarian division, has crippled the legislative process.^59^ However, key informant interviews suggest strong industry representation within the PHC, the main public body where tobacco control legislation has been debated, has been a key factor. In 2003 a comprehensive tobacco advertising ban was submitted by MPs, with the support of the Islamic Health Society, WHO, NTCP and Lebanese Consumer Association. The bill was discussed several times but rejected in August 2004. According to Saade, the basis for rejection was the Regie’s claim, supported by the MOF, of the likely economic impact to advertising (US $20 million annually) and employment. Instead, the Regie proposed restrictions on sales to those under 18 years old by removing billboards next to schools and sports clubs, not advertising in cinemas before 21:00 and broadcast media before 22:00, and banning samples.^60^ The ineffectiveness of industry proposals to reduce youth smoking has been described elsewhere.^61^

WHO’s adoption of the FCTC in 2005 prompted efforts in Lebanon to strengthen policy in line with the agreement. The PHC invited stakeholders to discuss advertising restrictions, health warnings, smoke-free places, sampling, and tar and nicotine labelling. Since 2004, the bill has undergone numerous reviews and discussion. It was also discussed by the Council of Ministers but no decision has been taken to date.

According to Majdalani, policy debate has been open and fair: “There were many sides and viewpoints…All discussed it [the bill] and gave their comments”. On the Regie he states, “Of course they tried to influence us and they gave their comments on the proposed law. Some we agree to and others we don’t”. For example, the Regie’s opposed banning packs of less than 10 cigarettes but this was rejected given their affordability to youth. On advertising, Majdalani believes economic interests need protecting:

There is no way we can do that [ban advertising] since we will be affecting the economic sector directly which…plays a major part in the Lebanese economy….in another sense this law does not do the job since fighting smoking is a bigger and wider social issue.

Najjar blamed tobacco control advocates for the lack of progress: “Be it either WHO or NGO’s [non-governmental organisations] or syndicate of doctors or any related to any other issue related to tobacco control, they are extreme in their thinking…and that is the reason why, in my opinion, the subject is not being effectively dealt with”. He advocated gradual change:

I don’t want to call it extremism. I want to call it immaturity. You cannot deal with a subject of this kind except gradually. I know from my work, as a strategist, the strategist to reach his objectives… builds a plan. He does not bump his head on the wall and say either my way or nothing.

This was echoed by Salam: “We are not against this at all. We are for regulation but it has to be done slowly and progressively”.

According to Mirna Wakid, President of the Lebanese Pulmonary Association, however, representation on the PHC was skewed, with industry interests outweighing public health advocates:

I was in total shock because… [Majdalani] got people together who are essentially against the project proposal altogether. The idea of having these people in the meeting! …I don’t understand how he got people who have conflicting interests.…All the people attending did not know what they were talking about. No one read or examined their documents. And at the same time you have the opposing camp, all opposing and not convinced with anything in tobacco control….

Because in their argument they were negative the whole time, they said this would not work, this we would not do…they had the right to veto things which is not asked from them or requested from them, since this is an experts meeting. Many barriers were put up. (Interview; Beirut, 13 June 2006)

The role of vested interests is echoed by MP and PHC member Ismail Sukkarieh: “The parliamentary members are made up of sects…you want someone to legislate… [and] even if they legislate you want them to implement…they will not control and implement against the interests of their own sects or leadership” (interview; Beirut, 17 April 2006). Former Prime Minister El Hoss describes, “Possible opponents are the MPs from the region where tobacco is grown. These members represent whole regions in the South whose living is from the income they get from selling tobacco” (interview; Beirut, 29 March 2006). Such livelihoods have been seriously affected by instability since 2006, with some fields affected by unexploded ordinance.^62^ For Zuhier Berro, President of the Lebanese Consumer Association, “gradual” change has benefited industry tactics:

We agreed to a programme but we did not agree on the length of the programme….what is happening is that year after year there is delay…

Even though it is true that we did not see or face sharp opposition to the law by companies, what is happening practically and factually is that until now no law was issued even though big sections of it have been proposed for many years…the opposition is not direct but is happening indirectly under the table. (Interview; Beirut, 20 March 2006)

This strategy has been particularly effective in a country already hampered by chronically weak governance. As Mroeh describes, “A weak political system will always do compromises and be under the influence of the lobbyist”.

## DISCUSSION

The weakness of tobacco control in Lebanon has been due, in large part, to effective industry tactics to weaken the content and scope of regulation and delay its adoption and implementation. Lebanon has been important as a “major influence in the Middle East…as trendsetters for consumer preferences”.^63^ Industry success has been due to several factors. First, strong alliances have been forged among TTCs, the Regie, associated industries (ie, advertising, distributors, farmers) and senior officials representing tobacco-dependent constituencies. These alliances, supported by the MEWG and META, have opposed bills at various stages of government discussion. Second, industry advocates have deployed economic arguments to oppose restrictions, backed by the vested interests of senior policy makers. Third, the industry has advocated ineffective voluntary codes to undermine binding regulations. Finally, the industry has benefited from weak governance caused by political and economic instability.

Ratification of the FCTC in December 2005 commits Lebanon to comprehensive tobacco control measures. These findings lead to several recommendations towards this goal. First, while a poorly functioning government can contribute to weak tobacco control, tobacco industry efforts to influence policymaking is more significant. The recent strengthening of environmental regulations in Lebanon through an active ministry and local NGOs,^64^ ^65^ suggests new policy initiatives can still be undertaken. Effective governance is an important, but not essential, factor in more effective health policy.^66^ ^67^ The public health community should work to publicly reveal industry strategies and counter its arguments. For example, the public health community should challenge longstanding economic rationales using evidence from the World Bank and other sources on taxation, privatisation, trade and foreign investment.

Second, industry-focused research should be supported to raise public awareness of its efforts to undermine legislation. Industry-initiated voluntary codes and corporate social responsibility programmes should be exposed. Increased public awareness of tobacco industry tactics in Lebanon should be part of the country’s ongoing struggle to strengthen governance.

Third, the undue influence of industry interests over tobacco control policies in Lebanon should be addressed through Article 5.3 of the FCTC which states that “Parties shall act to protect these policies from commercial and other vested interests of the tobacco industry in accordance with national law”.^68^ In fulfilling this obligation, the Lebanese government should draw on available guiding principles and guidelines for separating tobacco control policy from vested interests, such as rejecting voluntary codes, ensuring declarations of conflicts of interest, restricting industry groups from public bodies and transparent reporting of proceedings.

Fourth, the country currently lacks institutional capacity to take forward obligations. The public health community should act strategically, invigorating the NTCP with dedicated staff and resources. Coordination among the few organisations active in tobacco control, such as the Consumer Association, universities and medical associations, is essential. Regional and global links with like-minded organisations should be sought to draw on expertise available in other countries. Importantly, work must extend beyond the technical, to include engagement in political processes such as lobbying, advocacy and campaigning. Engagement with relevant ministries such as finance, economy, education, information, interior and social affairs is vital. Training in such methods should be provided through the WHO Tobacco Free Initiative and Framework Convention Alliance.

Finally, these efforts should be supported by the international community. Implementation of the FCTC, in particular, would benefit from effective reporting requirements by WHO, backed by appropriate public censure, that ensure member states fulfil their obligations. As Lebanese economist Marwan Iskandar describes, “unless the FCTC matters to the political interests of policy makers, they don’t give it attention. They sign up for a good image internationally and leave it to surveillance that takes place. International organisations rarely take the initiative to verify” (interview; Beirut, 14 April 2006). Sustained support for implementation by WHO, perhaps drawing comparative attention to regional performances, would help encourage policy makers to make tobacco control matter more in Lebanon.

**What this paper adds**

- Tobacco control in Lebanon remains weak despite ratification of the World Health organization (WHO) Framework Convention on Tobacco Control in 2005. There are no restrictions on direct advertising, promotion or sponsorship; no regulation of smoke-free environments; and one law requiring a very small and weak health warning label.
- To date there has been limited scholarly analysis of tobacco industry documents pertaining to Lebanon and the Middle East. This paper, supported by key informant interviews, provides the first detailed account of how the tobacco industry has exerted policy influence in Lebanon. Its findings provide important evidence to public health professionals, governments and advocates in their efforts to effectively regulate the tobacco industry.
- Increased public awareness of industry tactics in Lebanon is urgently needed as part of the country’s struggle to protect and promote public health, and to strengthen governance as a whole.
